# Clonal expansion and phenotypic alterations of TCR Vβ3^+^ T cells in juvenile-onset recurrent respiratory papillomatosis: implications for tumor-associated immunity and chemokine-mediated T-cell trafficking

**DOI:** 10.1128/jvi.01080-25

**Published:** 2026-06-02

**Authors:** Shilan Li, Wei Wang, Yun Peng, Guixiang Wang, Yue Xi, Shengcai Wang, Jing Zhao, Fengzhen Zhang, Hua Wang, Hongbin Li, Qingchuan Duan, Ting Long, Jingang Gui, Xin Ni, Jie Zhang

**Affiliations:** 1 Department of Otorhinolaryngology Head and Neck Surgery, National Center for Children’s Health, Beijing Children’s Hospital, Capital Medical University 12517, Beijing, China; 2 Department of Otorhinolaryngology, Eye Ear Nose and Throat Hospital of Fudan University 12478, Shanghai, China; 3 Laboratory of Tumor Immunology, National Center for Children’s Health, Beijing Pediatric Research Institute, Beijing Children’s Hospital, Capital Medical University 12517, Beijing, China; 4 Department of Nephrology, National Center for Children’s Health, Beijing Children’s Hospital, Capital Medical University 12517, Beijing, China; College of Agriculture & Life Sciences, University of Arizona, Tucson, Arizona, USA

**Keywords:** JORRP, TCR Vβ, clonal expansion, chemokine signaling, HPV, tumor microenvironment

## Abstract

Juvenile-onset recurrent respiratory papillomatosis (JORRP) is a rare, human papillomavirus (HPV)-driven pediatric disease characterized by recurring papillomas in the respiratory tract. The contribution of dysregulated T-cell immunity to disease severity remains poorly understood. This study included 97 JORRP patients and 124 controls. Due to sample limitations, downstream analyses, including flow cytometry, TCR, and RNA sequencing, were performed on distinct patient subsets (detailed in Methods). We identified a selective expansion of TCR Vβ3^+^ T cells, including expanded TRBV28 clonotypes, in patients with aggressive disease. These T cells exhibited heightened cytotoxicity and activation markers, and were enriched in papilloma tissues. Within the tumor microenvironment, gene expression profiling revealed upregulated chemokine signaling pathways (CCR2/CCL7, CXCR6/CXCL16) and key immunosuppressive markers, including PD-L1 and TGF-β1. Our findings demonstrate a tumor-specific clonal expansion and functional alteration of TCR Vβ3^+^ T cells in JORRP, suggesting these pathways as preliminary targets for further investigation.

**IMPORTANCE:**

Our study identifies a disease-specific T-cell signature in juvenile-onset recurrent respiratory papillomatosis (JORRP), marked by the clonal expansion of TCR Vβ3^+^ T cells with distinct TRBV28-dominant clonotypes. These T cells exhibit heightened cytotoxic potential and activation markers but appear functionally constrained within an immunosuppressive tumor microenvironment. Additionally, we discovered a dysregulated chemokine axis (CCR2/CCL7 and CXCR6/CXCL16), which likely facilitates T-cell recruitment, yet fails to sustain their antiviral activity. These findings provide mechanistic insights into why HPV-driven papillomas persist despite immune infiltration. Importantly, our work suggests that targeting TCR Vβ3^+^ T-cell responses and modulating key chemokine pathways could offer novel immunotherapeutic strategies to restore immune control and reduce disease recurrence in JORRP patients.

## INTRODUCTION

Juvenile-onset recurrent respiratory papillomatosis (JORRP) is a rare, benign neoplastic disorder of the respiratory mucosa in children, predominantly caused by human papillomavirus (HPV) types 6 and 11 (1). Although malignant transformation is infrequent, the recurrent growth of papillomas and their potential to obstruct the airway can severely compromise the quality of life in affected individuals (2). Evidence suggests that patients with JORRP exhibit impaired immune responses to HPV-6/11, largely due to limited interactions between T cells and papilloma-associated viral antigens within the tumor microenvironment (3, 4). Nonetheless, some adaptive immune responses, including the generation of HPV-specific antibodies, have been detected in these individuals (5, 6).

The immunological profile of JORRP patients reflects a complex and dysregulated immune landscape. Specific human leukocyte antigen (HLA) alleles, such as *HLA-DRB1* and *HLA-DQB1*, have been associated with disease severity (7, 8). Moreover, many of the immunological abnormalities described in JORRP, such as skewed Th1/Th2 balance and elevated Th2 cytokines, have been observed systemically in peripheral blood (9). In contrast, local studies of papilloma tissue have revealed reduced antigen presentation, immature Langerhans cells, and increased regulatory T-cell infiltration (10–12). These findings indicate that the immune dysfunction characteristic of JORRP may impede effective viral clearance and contribute to disease recurrence. Further investigation into specific immune cell subsets and their underlying molecular mechanisms may yield valuable insights for therapeutic intervention (13).

A growing area of interest in JORRP research is the role of tumor-specific T cells in anti-tumor immunity. The diversity and specificity of T-cell receptors (TCRs) enable a subset of T cells to recognize tumor-associated antigens and mediate targeted immune responses (14, 15). However, only a small fraction of these cells infiltrates tumors and exhibits functional anti-tumor activity. Recent advances in single-cell sequencing and immune repertoire profiling have facilitated detailed investigations into the phenotype and molecular features of tumor-specific T cells (16, 17). Elucidating the behavior of tumor-specific TCR-Vβ in JORRP may uncover novel mechanisms of immune evasion and identify potential targets for immunotherapeutic strategies.

## RESULTS

### Gini-TCR index skewing of T-cell subsets in JORRP

**Fig 1:**
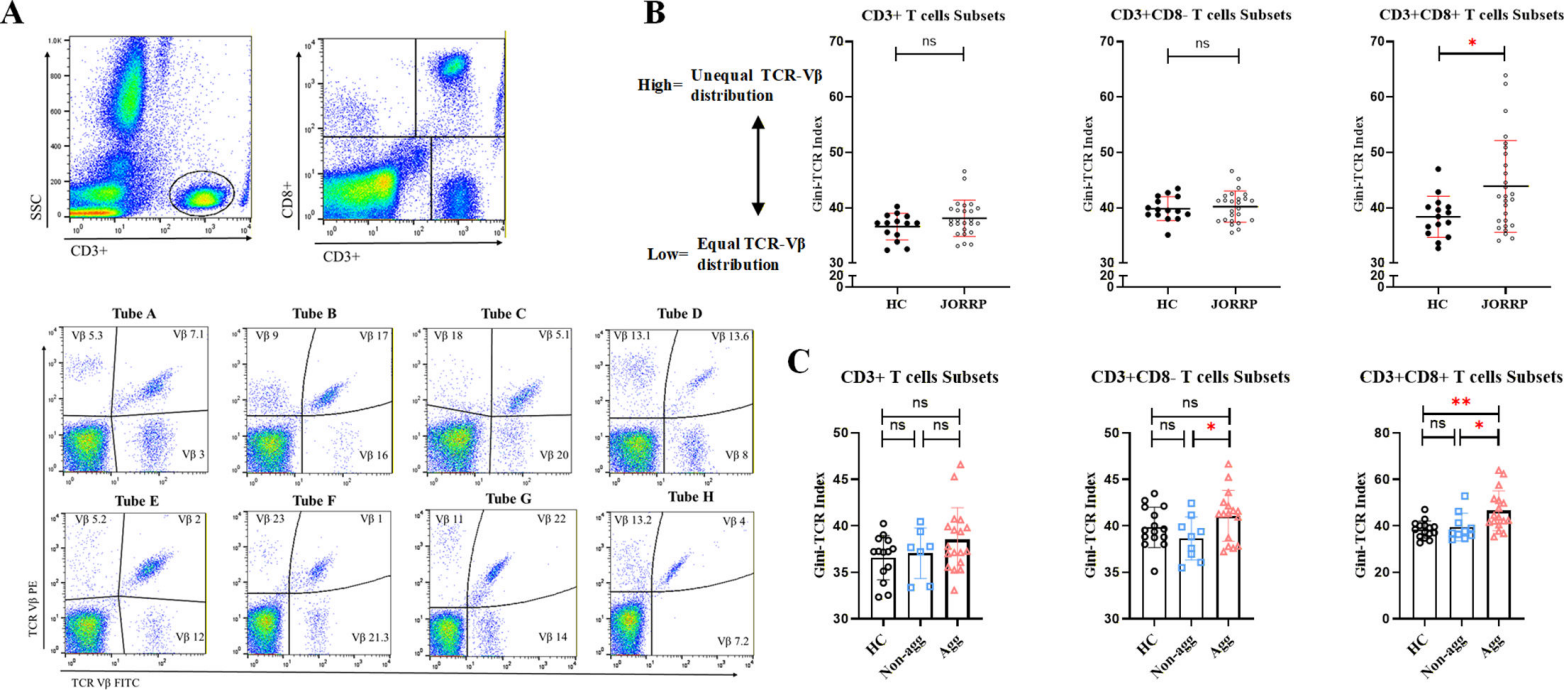
Flow cytometric analysis of T cell receptor (TCR) Vβ usage and the Gini-TCR index for different T cell subsets in JORRP. (**A**) Gating strategy for the detection of T cell receptor (TCR) Vβ usage in peripheral blood from Ctrl (*n* = 19) and JORRP patients (*n* = 36) was analyzed by flow cytometry. (**B**) Gini-TCR index for different T cell subsets between HC and JORRP patients. Significance indicated by *P < 0.05, using Student’s *t*-test or Mann-Whitney U test. (**C**) Gini-TCR index for different T cell subsets comparing HC, Agg (*n* = 23), and Non-agg (*n* = 13) patients. Significance indicated by *P < 0.05, **P < 0.01, using one-way ANOVA or Kruskal-Wallis H test. ns: no significance. Each dot represents an independent individual.

Flow cytometric analysis was performed to assess TCR-Vβ usage in CD3^+^, CD8⁻, and CD8^+^ T-cell subsets in patients with JORRP and healthy controls (HC) (Fig. 1A). The Gini-TCR index, which quantifies repertoire skewing within T-cell populations, revealed notable differences across groups. No significant differences were observed in the Gini-TCR index for CD3^+^ or CD8⁻ T cells between JORRP and HC groups (Fig. 1B). In contrast, CD8^+^ T cells from JORRP patients exhibited significantly higher Gini-TCR index values than those from HC (*P* < 0.05) (Fig. 1B), indicating increased repertoire skewing. Further stratification of JORRP patients into aggressive and non-aggressive subgroups showed that CD8^+^ T cells from the aggressive subgroup displayed significantly elevated Gini-TCR index values relative to both HC (*P* < 0.01) and non-aggressive patients (*P* < 0.05) (Fig. 1C). Similarly, CD8⁻ T cells from aggressive patients exhibited significantly greater Gini-TCR skewing compared with those from the non-aggressive subgroup (*P* < 0.05) (Fig. 1C). No significant differences in Gini-TCR index were detected in CD3^+^ T-cell subsets across groups (Fig. 1C). Collectively, these findings suggest a potential association between TCR repertoire contraction, particularly within CD8^+^ and CD8⁻ T-cell subsets, and increased disease severity in JORRP.

### Differences in TCR Vβ phenotypes between JORRP patients and healthy controls

**Fig 2:**
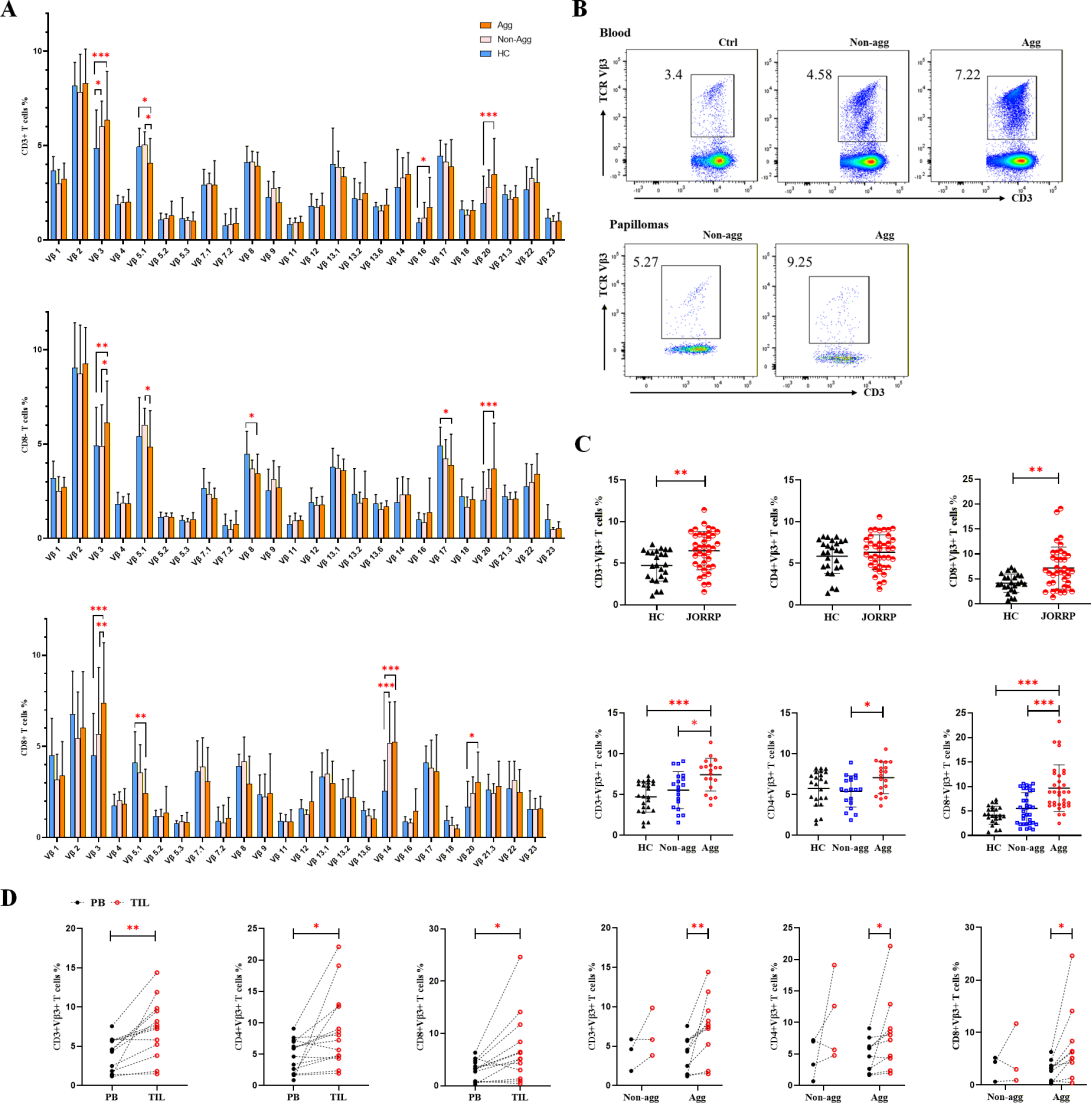
Flow cytometric analysis of the T cell receptor (TCR) Vβ repertoire and TCR Vβ3 usage in JORRP. Statistical comparisons were performed among all groups shown in the figure, with only statistically significant results displayed. (**A**) Flow cytometric analysis of the TCR Vβ repertoire in peripheral blood lymphocytes from JORRP patients (*n* = 36), including aggressive (Agg, *n* = 23) and non-aggressive (Non-agg, *n* = 13), compared to healthy controls (HC, *n* = 19). Significance indicated by *P < 0.05, **P < 0.01, ***P < 0.001, using one-way ANOVA or Kruskal-Wallis H test. (**B**) Gating strategy for detecting TCR Vβ3 in peripheral blood and papillomas by flow cytometry. Numbers adjacent to outlined areas indicated the percentage of cell subsets. (**C**) Flow cytometric analysis of TCR Vβ3+ T cell proportions in CD3+, CD4+, and CD8+ subsets in peripheral blood from JORRP (*N* = 39) versus HC (*n* = 36), and between Agg (*n* = 19) and Non-agg (*n* = 20) groups. Significance indicated by *P < 0.05, **P < 0.01, ***P < 0.001, using Student’s *t*-test or Mann-Whitney U test (JORRP vs HC), using one-way ANOVA or Kruskal-Wallis H test (HC, Non-agg vs. Agg). (**D**) Flow cytometric analysis of TCR Vβ3+ T cells proportions within CD3+, CD4+, and CD8+ subsets in papillomas (*N* = 14) and paired peripheral blood from Agg (*n* = 10) and Non-agg (*n* = 4) groups. Significance indicated by *P < 0.05, **P < 0.01, using paired *t*-test.

Flow cytometric analysis was performed to assess 24 TCR Vβ subsets in peripheral blood lymphocytes from patients with JORRP, including aggressive (*n* = 22) and non-aggressive (*n* = 14) cases, and HC (*n* = 19). In the aggressive group, significantly increased proportions of Vβ3^+^ and Vβ20^+^ T cells were observed across CD3^+^ (both *P* < 0.001), CD8⁻ (*P* < 0.01 and *P* < 0.001), and CD8^+^ (*P* < 0.001 and *P* < 0.05) subsets (Fig. 2A).

To validate these findings, we conducted flow cytometric analysis of Vβ3^+^ and Vβ20^+^ T cells in an independent cohort. Comparisons among aggressive (*n* = 20) and non-aggressive (*n* = 19) JORRP patients and HC (*n* = 36) confirmed a significant elevation of Vβ3^+^ T cells in the aggressive group across CD3^+^ (*P* < 0.001), CD4^+^ (*P* < 0.05), and CD8^+^ (*P* < 0.001) subsets, relative to both non-aggressive patients and HC (Fig. 2C). In contrast, non-aggressive JORRP patients exhibited no significant differences compared to HC, suggesting a stronger association between elevated Vβ3^+^ T cells and aggressive disease progression.

Given the marked increase in circulating Vβ3^+^ T cells in children with aggressive JORRP, we further examined their presence in local papilloma tissues. A paired analysis of peripheral blood and papillomas from non-aggressive (*n* = 4) and aggressive (*n* = 10) patients revealed significantly higher proportions of Vβ3^+^ T cells in the aggressive group within CD3^+^ (*P* < 0.01), CD4^+^ (*P* < 0.05), and CD8^+^ (*P* < 0.05) subsets from papillomas, compared to their matched peripheral blood samples (Fig. 2D). These findings suggest a preferential localization or enrichment of Vβ3^+^ T cells in papilloma tissues of patients with aggressive disease, further implicating this subset in disease severity.

**Fig 3:**
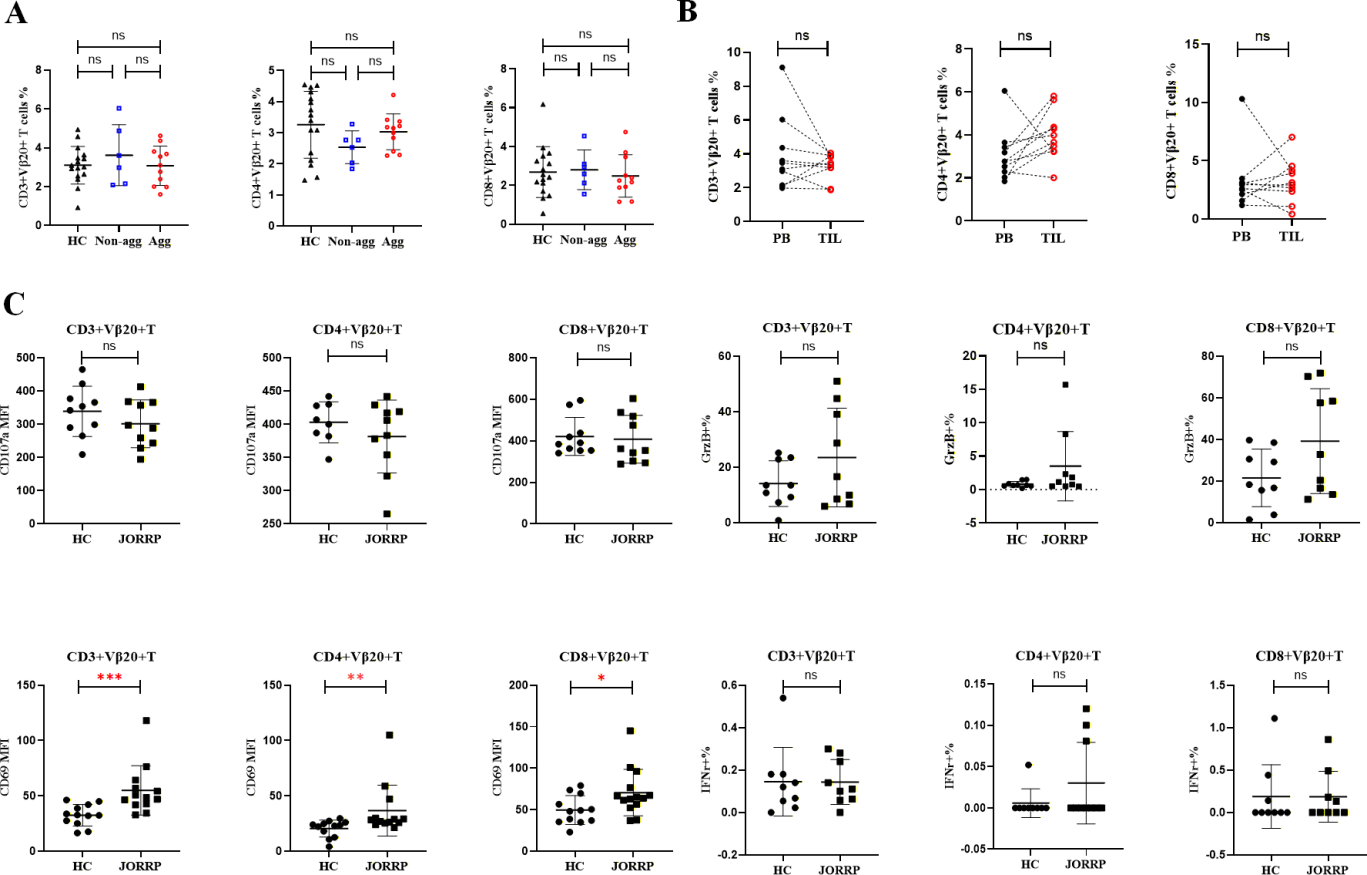
Flow cytometric analysis of the frequency and functional markers of TCR Vβ20+ T-cell subsets in JORRP. (**A**) Bar graphs depict the percentage of CD3+ Vβ20+, CD4+ Vβ20+, and CD8+ Vβ20+ T cells in aggressive (Agg, *n* = 12), non-aggressive (Non-agg, *n* = 5) JORRP, and Healthy Control (HC, *n* = 16) from peripheral blood. (**B**) Flow cytometric analysis of TCR Vβ20+ T cells proportions in CD3+, CD4+, and CD8+ subsets from papillomas (*n* = 10) and paired peripheral blood of JORRP patients. (**C**) Expression levels of CD107a, CD69, GrzB, and IFN-γ in TCR Vβ20+ T cells from peripheral blood of HC and JORRP patients are shown, highlighting differences in functional markers. *P < 0.05, **P < 0.01, ***P < 0.001. ns: not significant.

In contrast, flow cytometric analysis of Vβ20^+^ T cells in peripheral blood and papillomas (Fig. 3) revealed no statistically significant differences between JORRP patients (aggressive and non-aggressive) and HC. Due to the lack of association, Vβ20^+^ T cells were excluded from subsequent analyses. This enabled a focused investigation of TCR Vβ subsets, such as Vβ3^+^, that demonstrated stronger correlations with JORRP progression and clinical severity.

### Correlation analysis of TCR Vβ3^+^ T cells with clinical parameters and HPV genotype

**Fig 4:**
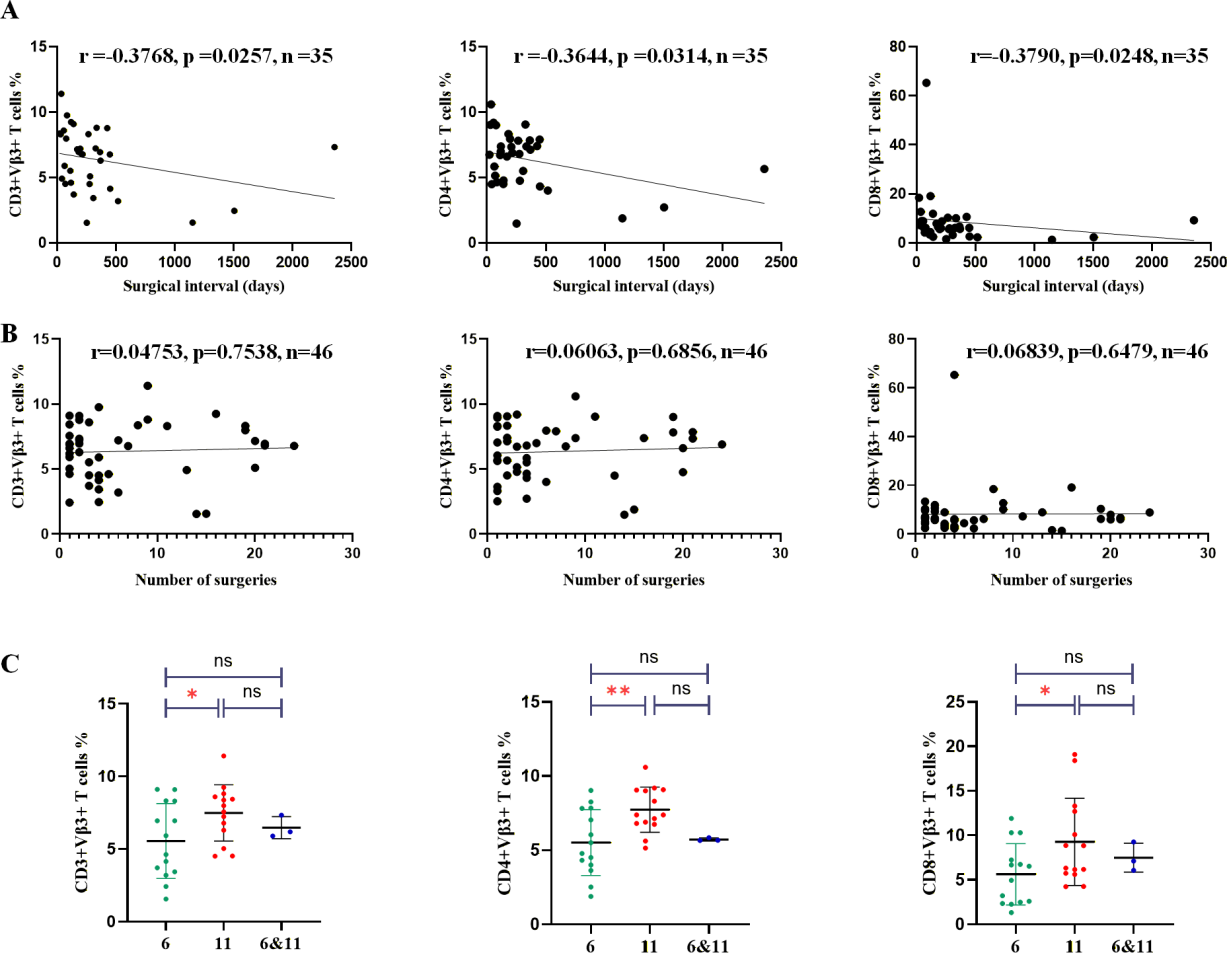
Relationship between TCR Vβ3+ T cells and clinical factors related to disease severity in JORRP patients. (**A**) Correlation between the proportion of CD3+, CD4+, and CD8+ TCR Vβ3+ T cells from peripheral blood in JORRP (*n* = 39) and the surgical interval (days). (**B**) Correlation between the proportion of CD3+, CD4+, and CD8+ TCR Vβ3+ T cells from peripheral blood in JORRP (*n* = 39) and the number of surgeries at the time of sampling. Pearson correlation coefficients (r) and exact P-values shown in panels A and B were determined using Pearson correlation analysis. (**C**) Statistical analysis of the proportion of CD3+, CD4+, and CD8+ TCR Vβ3+ T cells in peripheral blood from JORRP patients with different HPV types. Significance indicated by *P < 0.05, **P < 0.01, using one-way ANOVA test or Kruskal-Wallis H test. ns, no significance.

We next assessed the correlation between the frequency of TCR Vβ3^+^ T cells in peripheral blood and key clinical parameters of JORRP, including surgical interval, number of surgeries, and HPV genotype. The proportion of Vβ3^+^ T cells within CD3^+^, CD4^+^, and CD8^+^ subsets exhibited a significant negative correlation with surgical interval (Fig. 4A), indicating that patients with elevated Vβ3^+^ T cell frequencies required more frequent surgical interventions. Furthermore, analysis of the total number of surgeries revealed no strong correlation with Vβ3^+^ T cell proportions across any subset (Fig. 4B).

Previous studies have established an association between HPV11 infection and increased disease severity in JORRP (18–21). In the present study, we characterized the distribution of TCR Vβ3^+^ T cells across different HPV genotypes. Our analysis revealed that patients infected with HPV11 harbored a significantly higher proportion of Vβ3^+^ T cells within CD3^+^ (*P* < 0.05), CD4^+^ (*P* < 0.01), and CD8^+^ (*P* < 0.05) subsets compared to those with HPV6 (Fig. 4C). These findings suggest that the increased frequency of peripheral Vβ3^+^ T cells may be associated with HPV11 infection.

### Functional alteration of TCR Vβ3^+^ T cells in aggressive JORRP patients

**Fig 5:**
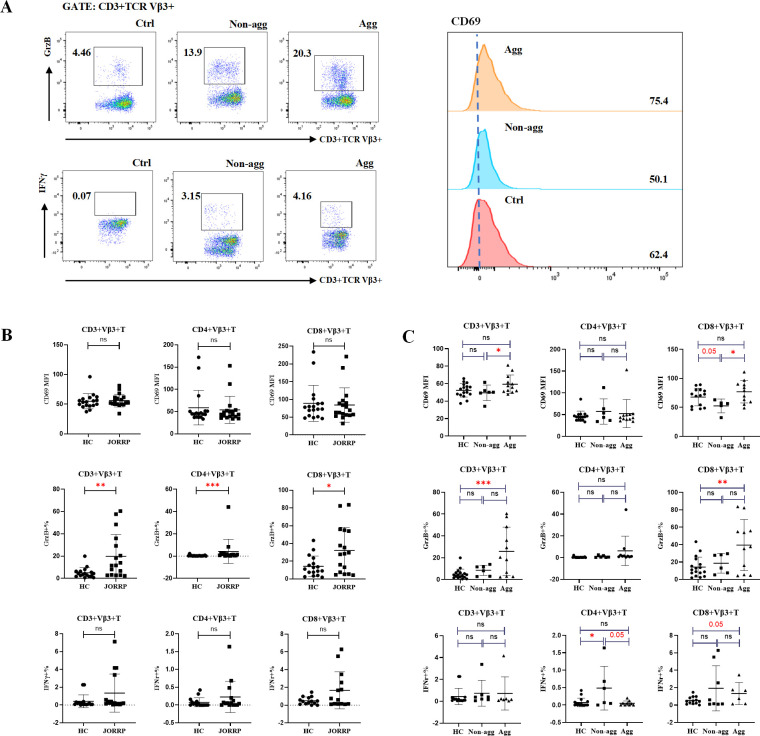
Function of TCR Vβ3+ T cells in peripheral blood of JORRP patients. (**A**) Flow cytometric gating strategy for GrzB and IFN-γ, along with the analytical approach for assessing CD69 expression in CD3+ TCR Vβ3+ blood cells from healthy controls (HC, *n* = 26), non-aggressive (Non-agg, *n* = 9), and aggressive (Agg, *n* = 12) JORRP patients. Numbers indicate the percentage of positive cells within each gate. (**B**) CD69, GrzB, and IFN-γ expression in CD3+, CD4+, and CD8+ TCR Vβ3+ T cells from HC and JORRP patients. Significance indicated by *P < 0.05, **P < 0.01, ***P < 0.001, using Student’s *t*-test or Mann-Whitney U test. (**C**) CD69, GrzB, and IFN-γ expression in CD3+, CD4+, and CD8+ TCR Vβ3+ T cells from HC, Non-agg, and Agg JORRP patients. Significance indicated by *P < 0.05, **P < 0.01, ***P < 0.001, using one-way ANOVA or Kruskal-Wallis H test. ns: no significance.

Flow cytometric analysis of peripheral blood from HC and patients with JORRP, including aggressive and non-aggressive subgroups, revealed distinct cytokine production and activation profiles in TCR Vβ3^+^ T cells (Fig. 5A through C). Expression of the early activation marker CD69 was significantly elevated in CD3^+^ and CD8^+^ Vβ3^+^ T cells from the aggressive group compared to the non-aggressive group (*P* < 0.05 for both), indicating heightened activation in aggressive cases (Fig. 5C). No significant differences were observed in CD107a expression among the groups (data both *P*＞0.05).

Granzyme B (GrzB) expression was significantly increased in CD3^+^, CD4^+^, and CD8^+^ Vβ3^+^ T cells in JORRP patients compared to HC (Fig. 5B), with the highest levels observed in the aggressive group (*P* < 0.001 for CD3^+^; *P* < 0.01 for CD8^+^; Fig. 5C), supporting enhanced cytotoxic potential in these individuals. With respect to interferon-γ (IFN-γ), CD4^+^ Vβ3^+^ T cells from the non-aggressive group exhibited significantly higher expression compared to HC (*P* < 0.05; Fig. 5C). In CD8^+^ Vβ3^+^ T cells, IFN-γ expression was elevated in the aggressive group relative to HC, with values approaching statistical significance (*P* = 0.05; Fig. 5C). Stratification by HPV genotype showed no significant differences in these functional markers on Vβ3^+^ T cell subsets between HPV6- and HPV11-infected patients (Fig. S1).

Collectively, these findings suggest that GrzB-mediated cytotoxicity is a key feature of JORRP pathogenesis, particularly in aggressive disease. In addition, increased CD69 expression and differential IFN-γ production in Vβ3^+^ T cells may reflect immune activation and modulation, further contributing to disease severity in aggressive JORRP.

### Transcriptional profiling of CD3^+^TCR Vβ3^+^ T cells in JORRP patients reveals altered immune and chemokine pathways

**Fig 6:**
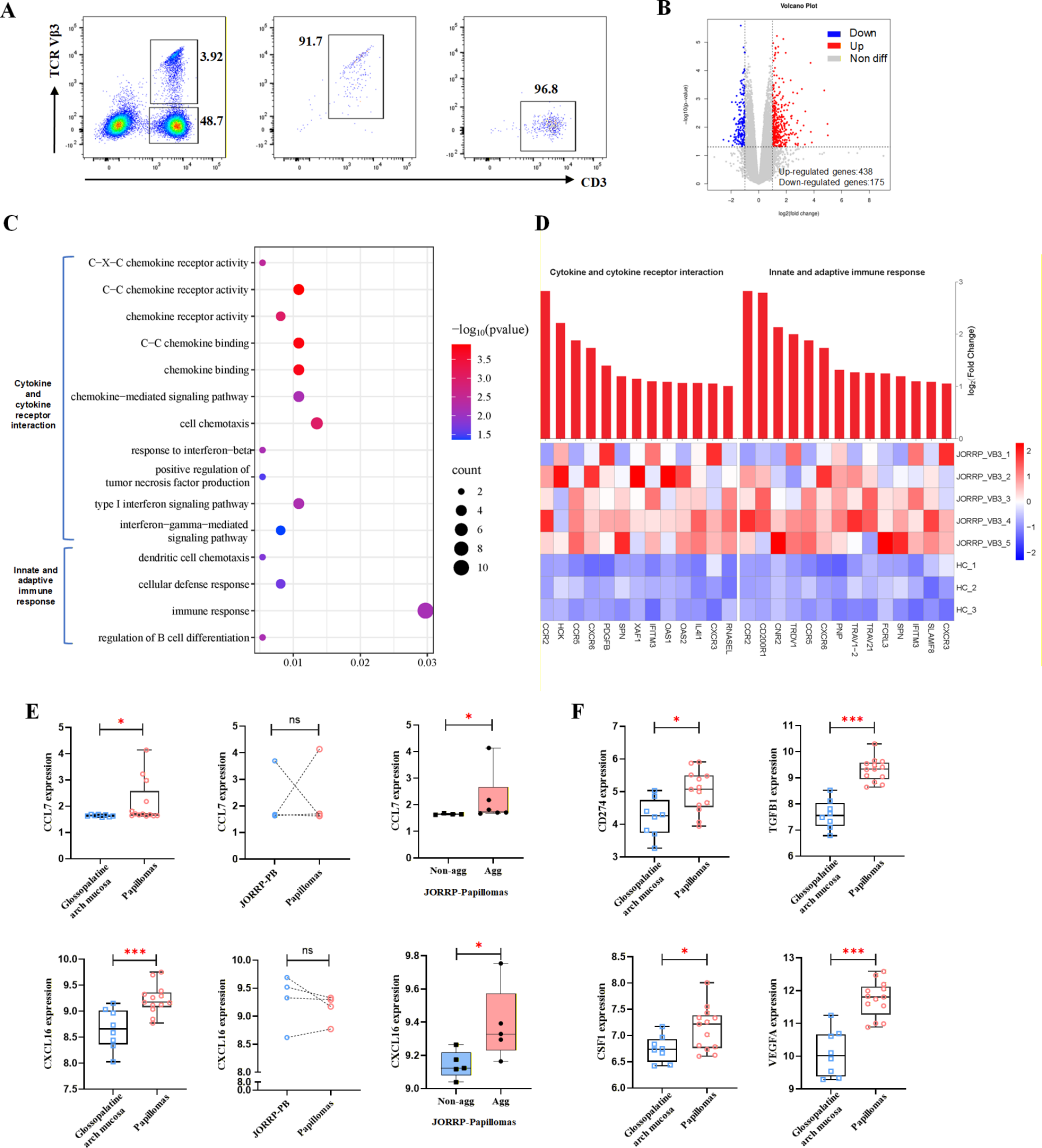
Differential gene expression reveals distinct transcriptional profiles and functional changes in CD3+ TCR Vβ3+ T Cells in JORRP. (**A**) Gating strategy for sorting CD3+ TCR Vβ3+ T cells in peripheral blood and purity analysis of the sorted cells from a representative JORRP patient. Numbers indicate percentages within gates. (**B**) Volcano plot illustrating the differentially expressed genes (DEGs) in CD3+ TCR Vβ3+ T cells sorted from JORRP (*N* = 5) compared with Health Control (HC, *n* = 3). Red dots represent upregulated genes, and blue dots indicate downregulated genes, with significant changes (*P* < 0.05) between the two groups. (**C**) Gene Ontology (GO) enrichment analysis of DEGs in circulating CD3+ TCR Vβ3+ T cells, comparing JORRP with Health Control in two aspects. (**D**) Heatmap of upregulated (red) and downregulated (blue) genes in pathways of Fig. 5C in circulating CD3+ TCR Vβ3+ T cells in JORRP versus Health Control. (**E**) Box-plot analysis comparing the expression levels of chemokine genes (e.g., CCL7 and CXCL16) in papillomas from non-aggressive (*n* = 6) and aggressive (*n* = 7) JORRP, as well as in glossopalatine arch mucosa (*n* = 8) and paired JORRP bloods (*n* = 4). (**F**) The box plots showing the expression difference of suppressive cytokine genes in papillomas (*n* = 13) and glossopalatine arch mucosa (*n* = 8) as measured by RNA microarray. (**E** and **F**) Significance indicated by *P < 0.05, ***P < 0.001, using Student’s *t*-test or Mann-Whitney U test. ns: not significant.

Following the isolation of CD3^+^TCR Vβ3^+^ T cells from the peripheral blood of JORRP patients and HC (Fig. 6A), RNA sequencing and Gene Ontology (GO) pathway analysis revealed substantial transcriptional alterations in JORRP patients relative to HC. A total of 438 genes were upregulated (red), and 175 genes were downregulated (blue) in JORRP-derived T cells (Fig. 6B), indicating significant changes in gene expression profiles.

GO enrichment analysis of the upregulated differentially expressed genes (DEGs) demonstrated significant activation of pathways related to cytokine-cytokine receptor interactions, as well as both innate and adaptive immune responses in CD3^+^TCR Vβ3^+^ T cells from JORRP patients compared with HC (Fig. 6C). A corresponding heatmap (Fig. 6D) illustrates the expression patterns of genes associated with these enriched pathways, showing distinct clustering between patient and control groups. Notably, chemokine receptor genes such as *CCR2*, *CCR5*, and *CXCR6* were markedly upregulated in CD3^+^TCR Vβ3^+^ T cells from JORRP patients, suggesting enhanced immune activation and potential recruitment of these cells to the tumor microenvironment (Fig. 6D). Among the chemokine ligands, *CCL7*, a ligand of *CCR2*, was upregulated in papilloma tissues compared with glossopalatine arch mucosa from children with tonsil hypertrophy and exhibited higher expression in aggressive JORRP cases (Fig. 6E). *CXCR6*, a receptor critical for the migration of resident memory T cells, was similarly upregulated, along with its ligand *CXCL16*, which showed significantly elevated expression in papillomas relative to glossopalatine arch mucosa (*P* < 0.001), with particularly increased levels in the aggressive group (*P* < 0.05) (Fig. 6E). These findings highlight the chemokine-driven dynamics of immune cell trafficking in JORRP and suggest potential therapeutic targets within this signaling axis.

In addition, gene expression analysis revealed a significant upregulation of immune suppression–associated genes in papilloma tissues compared to glossopalatine arch mucosa. Specifically, *CD274* (PD-L1), *TGFB1*, *CSF1*, and *VEGFA* showed higher mRNA expression levels in papillomas (Fig. 6F). Within the tumor microenvironment, PD-L1 and CSF-1 are critical mediators of immune evasion (22), while TGF-β and VEGFA have been implicated in the recruitment of immunosuppressive cell populations, including regulatory T cells, thereby contributing to an immunosuppressive milieu (23, 24).

### TRBV28 clonotype expansion in JORRP patients based on TCR sequencing of peripheral blood and papilloma tissues

**Fig 7:**
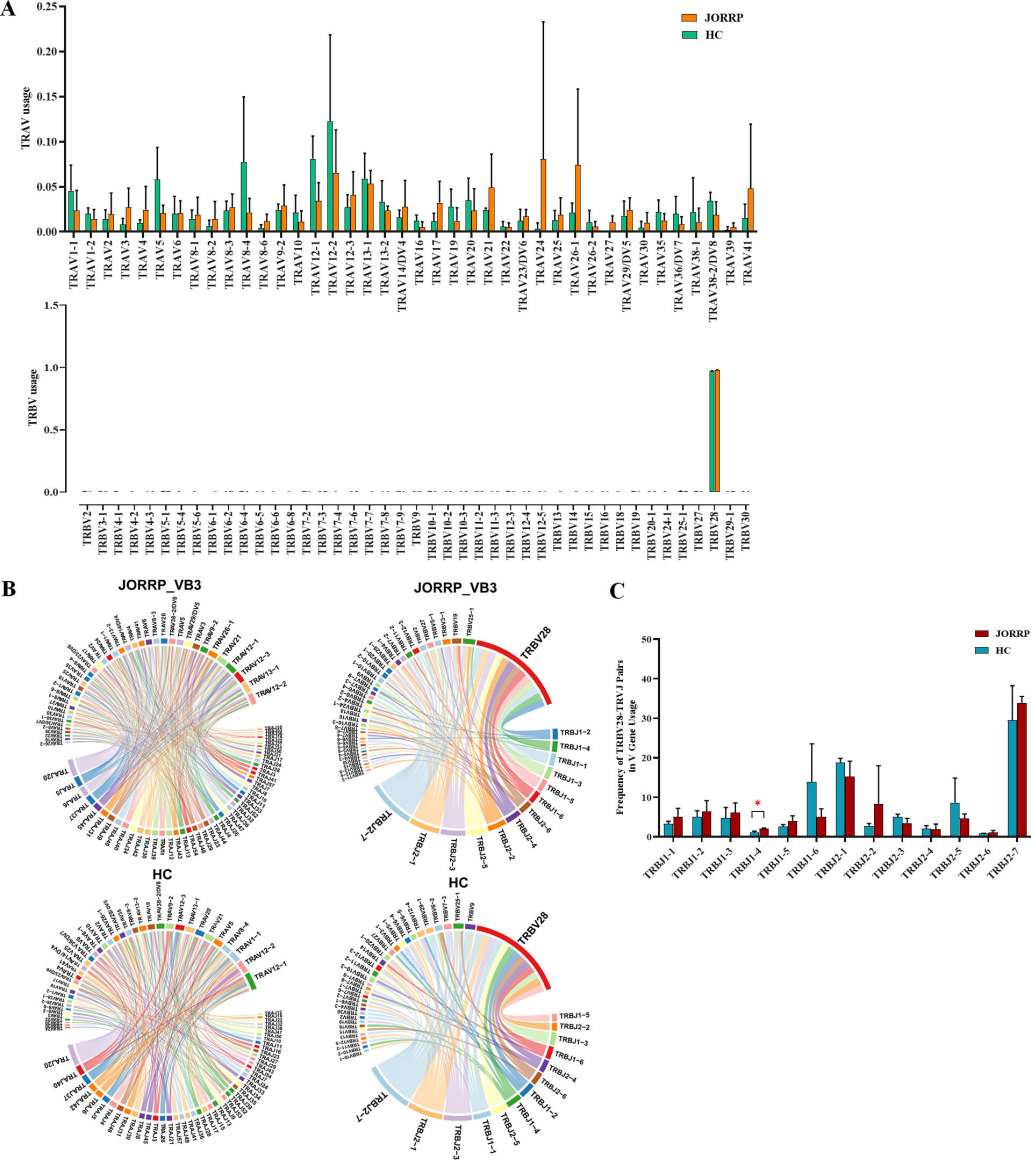
The biased TCR use in circulating CD3+ TCR Vβ3+ T cells. (**A**) TRAV (upper panel) and TRBV (lower panel) usage of selected CD3+ TCR Vβ3+ T cells in JORRP (*n* = 5) and healthy control (HC, *n* = 3). (**B**) Circos plots showing the most frequent TRAV–TRAJ and TRBV–TRBJ pairs in CD3+ TCR Vβ3+ T cells from JORRP (*N* = 5) and healthy controls (*n* = 3). (**C**) The frequency of the TRBV28_TRBJ pairing in JORRP (*n* = 5) compared to healthy controls (*n* = 3). Statistical comparisons were performed among all groups shown in the figure, with only statistically significant results displayed. Significance indicated by *P < 0.05, using Student’s *t*-test.

RNA sequencing and TCR analysis revealed distinct alterations in the TCR repertoire of CD3^+^TCR Vβ3^+^ T cells in patients with JORRP compared to HC. Analysis of TRBV gene usage revealed that TRBV28 was the dominant segment in both JORRP and HC cohorts (frequency: 0.9710 vs. 0.9817; Fig. 7A). Circos plots of TRAV–TRAJ and TRBV–TRBJ pairings indicated extensive clonal expansion within the T-cell population (Fig. 7B). Notably, the *TRBV28_TRBJ1-4* pairing was significantly more frequent in JORRP patients than in HC (*P* < 0.05), suggesting a potential role in the disease-specific immune response (Fig. 7C). However, due to limited data from the sequencing of sorted T cells, further in-depth analysis was not feasible at this stage. To address this limitation, high-throughput TCR sequencing was conducted on whole peripheral blood samples from JORRP patients and HC, as well as on papilloma tissues from JORRP patients. Clonotypes containing *TRBV28* were extracted for immune repertoire analysis and cross-group comparison.

**Fig 8:**
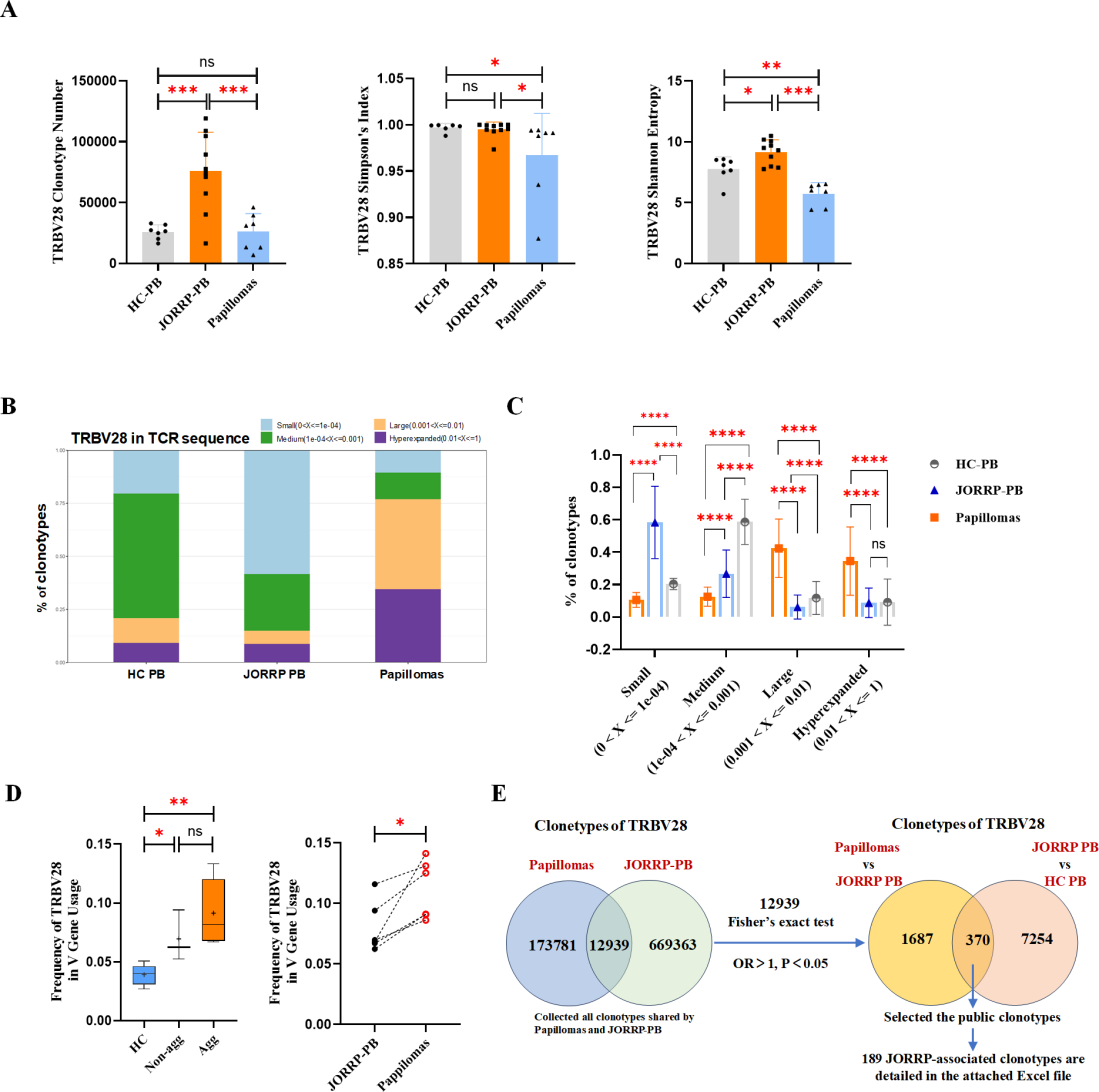
Features of the TRBV28 clonotypes from TCR sequencing in papillomas (*n* = 7), JORRP peripheral blood (JORRP-PB, *n* = 10), and healthy controls peripheral blood (HC-PB, *n* = 7). (**A**) Comparison of TRBV28 clonotype diversity among the three groups, measured by clonotype number, Simpson’s index, and Shannon entropy. Significance indicated by *P < 0.05, **P < 0.01, and ***P < 0.001, using one-way ANOVA test. (**B**) Distribution of TRBV28 clonal expansion frequencies by clone size. Clonotypes are categorized as small (0 < X ≤ 1e-4), medium (1e-4 <X ≤ 0.001), large (0.001 < X ≤ 0.01), and hyperexpanded (0.01 < X ≤ 1). (**C**) Proportion analysis of clonal types across the three groups. Hyperexpanded and large clonotypes are significantly more frequent in papillomas than in JORRP-PB and HC-PB (****P < 0.0001, two-way ANOVA). (**D**) TRBV28 gene expression is significantly higher in JORRP-PB, especially in the aggressive group, compared to HC-PB (*P < 0.05, **P < 0.01, using one-way ANOVA). TRBV28 gene expression is also markedly higher in papillomas than in paired JORRP-PB (*P < 0.05, using paired *t*-test). (**E**) Identification of disease-specific TRBV28 clonotypes in JORRP. The left Venn diagrams show overlapping clonotypes between papillomas and JORRP-PB. The right Venn diagrams display the intersection of Fisher’s exact test (OR > 1, *P* < 0.05) results from two comparisons: papillomas vs. JORRP-PB and JORRP-PB vs. HC-PB. Selecting the public clonotypes, expressed in at least two patients with ≥ 10 reads, were retained, resulting in 189 disease-specific clonotypes, detailed in Data Set S1.

Comprehensive analysis revealed a significantly higher *TRBV28* clonotype count in JORRP peripheral blood compared to both HC and papilloma tissues. In contrast, Simpson’s index and Shannon entropy were markedly reduced in papillomas (Fig. 8A), indicating lower diversity and increased dominance of specific clonotypes within the tumor microenvironment. Clonal expansion frequency analysis based on clonotype size (Fig. 8B and C) demonstrated a predominance of large and hyperexpanded *TRBV28* clonotypes in papilloma tissues, whereas peripheral blood from both JORRP patients and HC primarily contained small clonotypes, highlighting the localized clonal expansion in tumor tissue.

In our previous analysis of the full TRAV and TRBV genes, *TRBV28* expression was significantly elevated in both papillomas and PBMCs from JORRP patients compared to HC PBMCs (25). Expanding on these findings, we stratified patients into aggressive and non-aggressive groups. *TRBV28* clonotype usage was significantly elevated in both patient groups relative to HC (Fig. 8D). Although the aggressive group exhibited a higher frequency than the non-aggressive group, the difference did not reach statistical significance. Paired analysis further confirmed greater *TRBV28* clonotype expansion in papilloma tissues than in matched peripheral blood samples (Fig. 8D). These findings genetically validate that TCR Vβ3^+^ T cells are tumor-associated and disease-relevant in JORRP.

Finally, cross-group comparisons identified 189 public *TRBV28* clonotypes, defined by their presence in at least two patients with a read count ≥10 (Fig. 8E). These clonotypes were significantly enriched in papilloma tissues relative to peripheral blood, as determined by Fisher’s exact test, and were also more highly expressed in JORRP peripheral blood compared to HC. To further characterize these clonotypes, we interrogated their CDR3 beta amino acid sequences and V/J gene pairings against publicly available TCR databases, including VDJdb (https://vdjdb.cdr3.net/), McPAS-TCR (http://friedmanlab.weizmann.ac.il/McPAS-TCR/), and the Immune Epitope Database (IEDB; https://www.iedb.org/), and identified three exact matches. The CDR3 beta sequence CASSLSTDTQYF (TRBV28, TRBJ2-3) and CASSRDSSYEQYF (TRBV28, TRBJ2-7) were found in McPAS-TCR, associated with colorectal cancer (26). Another match for CASSLTGNYGYTF (TRBV28, TRBJ1-2) was linked to *Mycobacterium tuberculosis* infection (27) in CD4+ peripheral blood mononuclear cells. No specific antigens, epitopes, or MHC restrictions were reported for these matches, and no additional clonotypes from our list matched entries in any of the databases, including for HPV-related antigens. Detailed information on these clonotypes is provided in Data set S1. These shared tumor-associated clonotypes may play a key role in the immune response to HPV-induced papillomas in JORRP.

## DISCUSSION

In this study, we first provided a global overview of TCR Vβ usage in peripheral T cells from patients with JORRP, based on the classification criteria of Wei et al. (28) (Fig. 1 and 2). Our findings revealed that TCR Vβ3^+^ T cells represent the predominant intra-papilloma T-cell subset responding to HPV6/11 infection. TCR sequencing further identified *TRBV28* as the principal genotype within the Vβ3^+^ population, with clear evidence of clonal expansion in the papillomas of patients, particularly those with aggressive disease (Fig. 7 and 8). Functional profiling of these TCR Vβ3^+^ T cells revealed heightened activation and cytotoxic potential in aggressive JORRP, as indicated by the upregulation of GrzB and interferon-gamma (IFN-γ) (Fig. 7). In addition, chemokine signaling pathways, including the CCR2/CCL7 and CXCR6/CXCL16 axes, were upregulated and may facilitate the recruitment and retention of these T cells at tumor sites, thereby representing potential therapeutic targets.

Although TCR Vβ3^+^ T cells were identified as a likely antigen-specific subset in JORRP, their inability to control disease progression remains incompletely understood. Elevated expression of immunosuppressive factors such as *CD274* (PD-L1), *TGFB1*, *CSF1*, and *VEGFA* in papillomas suggests that these T cells operate within an inherently immunosuppressive microenvironment. Previous studies have also demonstrated an increased frequency of regulatory T cells (Tregs) in papillomas from patients with aggressive JORRP, with these Tregs secreting immunosuppressive cytokines including IL-4, IL-10, and TGF-β (10, 11). Furthermore, the presence of immature Langerhans cells, characterized by diminished antigen-presenting capacity, has been frequently observed in the papilloma tissues of these patients (12). Additionally, immune evasion mechanisms, such as downregulation of transporter associated with antigen processing 1 (TAP-1) and major histocompatibility complex (MHC) class I expression, have been documented in papilloma biopsies and primary cell explants from JORRP patients (29).

Our findings align with and extend previous work on immune dysregulation in recurrent respiratory papillomatosis (RRP) (30–32). Consistent with earlier studies, we observed significant TCR repertoire skewing and clonal expansion of specific T-cell subsets, suggesting an ongoing antigen-specific immune response against papilloma-associated antigens. Such clonal expansions are reminiscent of findings in other antigen-driven conditions, including Rasmussen encephalitis (33), type 2 diabetes (34), and hepatitis B virus (HBV)-associated hepatocellular carcinoma (35). The elevated cytotoxic activity of TCR Vβ3^+^ T cells, evidenced by increased GrzB and IFN-γ expression, also corroborates prior observations of hyperactivated T cells in HPV-associated diseases (36–38). Stratification by viral genotype revealed that while HPV11 drives greater Vβ3^+^ expansion, the cytotoxic and activation profiles of these cells do not differ significantly between HPV6 and HPV11 infection. This suggests Vβ3^+^ T cell hyperactivation is a conserved mechanism across viral subtypes, although our small sample size may limit the power to detect subtle differences. Collectively, these findings highlight the central role of this hyperactivated, yet dysregulated, T-cell response in RRP persistence and progression.

By applying advanced methodologies and a personalized analytical approach, we characterized the TCR repertoire and functional profiles of tumor-associated T cells in JORRP at high resolution. In contrast to earlier studies that relied primarily on flow cytometry or bulk sequencing to broadly classify T-cell subsets (39–41), our combined use of fluorescence-activated cell sorting (FACS), RNA sequencing, and high-throughput TCR sequencing enabled detailed characterization of hyperexpanded T-cell clonotypes. We identified *TRBV28* as the dominant clonotype among expanded TCR populations in papillomas and detected 189 public clonotypes enriched in papilloma tissue. To determine the pathological relevance of these clonotypes, we applied a stringent algorithm: only clones expressed in at least two JORRP patients with ≥10 reads and showing minimal or no expression in healthy controls were classified as “JORRP-specific.” Database searches (VDJdb, McPAS-TCR, and IEDB) identified matches for three sequences to colorectal cancer and *M. tuberculosis*, but no HPV-related antigens were found. The absence of known antigen matches suggests that most JORRP-specific clonotypes may recognize novel or uncharacterized epitopes that remain unidentified and uncataloged in public databases, particularly for low-risk HPV types (e.g., HPV6/11) associated with JORRP. The partial matches to sequences from unrelated pathologies, such as tumors and bacterial infections, imply convergent TCR usage, where similar CDR3 motifs arise independently in response to structurally analogous antigens across diverse immune contexts.

Previous studies have demonstrated the promise of TCR-engineered T cell (TCR-T) therapies for HPV-associated malignancies by targeting HPV-specific TCRs (42, 43). Our identification of expanded *TRBV28* clonotypes, particularly *TRBV28_TRBJ1-4* pairings, and their correlation with aggressive disease suggests that these TCRs may serve as promising candidates for such targeted approaches. However, given the limited size of our sequencing cohort, we acknowledge that these specific clonotypic findings remain preliminary. Whether TRBV28 represents a generalized feature of aggressive JORRP or a cohort-specific observation requires extensive validation in larger studies before establishing it as a definitive therapeutic target. Beyond specific clonotypes, the observed correlation between TCR repertoire skewing and disease severity underscores the potential of diversity metrics, such as the Gini index, as biomarkers for patient stratification and therapeutic monitoring. Such approaches are particularly relevant for precision immunotherapy, enabling real-time monitoring of TCR dynamics and identification of patients who may benefit from T cell-based interventions (44).

The CCR2/CCL7 and CXCR6/CXCL16 axes appear to play central roles in immune cell trafficking within the tumor microenvironment (45–48). CCR2 is expressed on monocytes, macrophages, and T cells, whereas CXCR6 is predominantly expressed on T and natural killer (NK) cells. Their ligands, CCL7 and CXCL16, respectively, facilitate immune cell migration in tumor contexts (49, 50). In our study, we observed significant upregulation of *CCR2* and *CXCR6* in peripheral TCR Vβ3^+^ T cells from JORRP patients, along with elevated *CCL7* and *CXCL16* expression in papilloma tissues. These findings suggest that CCR2/CCL7 may promote T-cell infiltration, while CXCR6/CXCL16 may enhance T-cell activation and persistence (51, 52). Their combined effects may potentiate T-cell recruitment and effector function in JORRP; however, mechanistic studies are needed to confirm these roles. Therapeutic modulation of these axes (e.g., via CCR2 or CXCR6 agonists) may represent a promising strategy for enhancing anti-tumor immunity in JORRP.

Despite these advances, several challenges remain in translating our findings into clinical applications. First, although we identified key TCR clonotypes and chemokine pathways, functional validation of these targets in preclinical and clinical models is warranted. Our focus on the CCR2/CCL7 and CXCR6/CXCL16 pathways, while informative, does not encompass the full spectrum of chemokine and immunosuppressive mechanisms present in the tumor microenvironment (51, 53). Second, although glossopalatine arch mucosa serves as a histologically comparable surrogate (squamous epithelium), potential site-specific immunological differences between the larynx and pharynx remain a limitation. Consequently, our comparative transcriptomic findings should be interpreted with this anatomical context in mind. Emerging technologies, such as single-cell transcriptomics and spatial mapping, may offer deeper insights into the interplay between TCR clonotypes and local immune architecture, ultimately improving our understanding of HPV-driven immune dysregulation (54, 55).

In conclusion, this study highlights the critical involvement of TCR Vβ3^+^ T cells in the immune alteration characteristic of JORRP. Although these cells exhibit clonal expansion, their apparent inability to control disease progression suggests the presence of immunosuppressive mechanisms within the tumor microenvironment that impair effective immune responses. The observed upregulation of chemokine signaling pathways, including CCR2/CCL7 and CXCR6/CXCL16, underscores the complex dynamics governing T-cell recruitment and retention within papilloma tissues. Future investigations should focus on elucidating the roles of immune checkpoint molecules, regulatory T cells, and other immunosuppressive components that may contribute to immune evasion in JORRP. In parallel, exploring specific TCR clonotypes or modulating chemokine-mediated signaling pathways may represent promising therapeutic avenues. Longitudinal analyses of the TCR repertoire in response to treatment could further inform personalized immunotherapeutic approaches for HPV-associated diseases. Collectively, these findings provide a foundation for future research aimed at restoring effective anti-viral immunity in JORRP and improving clinical outcomes for affected individuals.

## MATERIALS AND METHODS

### Study population and ethics

In total, 97 patients (a total of 112 peripheral blood samples and 41 papilloma samples) from China who were treated in the Otorhinolaryngology Department of Beijing Children’s Hospital from February 2015 to January 2024 were recruited to the study. Patients were identified through hospital registry systems and histopathological examination. The records of patients were reviewed by two laryngologists; 116 age- and race-matched healthy controls (HC) and 8 children with tonsil hypertrophy were also enrolled in this study during the same period. JORRP patients were categorized into Agg and Non-agg groups based on established clinical criteria. The disease was classified as aggressive if any of the following were observed (56): total procedures ≥ 10, procedure frequency ≥ 4 per year, distal involvement, or the presence of a tracheostomy tube.

Due to ethical constraints regarding pediatric blood collection (strictly limited to 1–2 mL per visit), the total blood volume obtained from a single draw was insufficient to perform the full suite of experimental assays simultaneously. Leveraging the recurrent nature of JORRP, which necessitates repeated hospital admissions, samples were collected from the same patients at different clinical time points to complete the various arms of the study. To ensure statistical rigor, the number of independent JORRP patients and control participants included in each specific assay, along with their respective clinical characteristics, is summarized in Table 1. For full transparency, a comprehensive mapping of individual patients across the different experimental arrays, including their longitudinal clinical data, is provided in Table S1.

**TABLE 1:** Clinical characteristics of independent patients and samples included in each assay^*a*^^,*b*^

Sample	Flow cytometry of 24Vβ	Flow cytometry of Vβ3			Flow cytometry of Vβ20		UID RNA-seq of TCR Vβ3+ T cell	RNA sequencing	TCR sequencing
		Proportions in blood	Papilloma vs. blood	Function	Proportion and function in blood	Papilloma vs. blood			
JORRP									
Blood samples of independent patients (*n*)	36	39	14	21	17	10	5	4	10
Papilloma samples (*n*)	0	0	14	0	0	10	0	13	7
Sex, female/male	17/19	13/26	7/7	5/16	4/13	0/10	2/3	7/6	6/5
Age (yr)	3.81 ± 3.05	4.73 ± 2.45	5.61 ± 4.03	4.34 ± 2.14	3.79 ± 2.16	3.43 ± 2.42	1.67 ± 0.79	3.99 ± 2.02	5.13 ± 3.30
Aggressive characteristics (agg/non-agg)	23/13	20/19	10/4	12/9	12/5	5/5	3/2	7/6	7/4
HPV type (6/11/6+11)	13/18/1, 4ND	14/14/3, 8ND	2/11/0, 1ND	10/11/0	8/8/0, 1ND	4/6/0	5/1/0	4/5/0, 4ND	1/7/2, 1ND
Control									
Blood samples of healthy control (*n*)	19	36	0	26	16	0	13	0	6
Glossopalatine arch mucosa of OSA patients (*n*)	0	0	0	0	0	0	0	8	0
Sex, female/male	7/12	13/23	–	4/22	2/14	–	7/6	4/4	4/2
Age (yr)	5.05 ± 4.29	4.67 ± 2.72	–	4.10 ± 2.31	3.60 ± 2.48	–	1.69 ± 0.63	5.44 ± 2.18	5.33 ± 3.61

^*a*^ agg, aggressive; non-agg, nonaggressive; ND, not determined; –, not applicable due to a sample size of zero (*n* = 0).

^*b*^ Some patients contributed multiple samples at different clinical time points; however, within each specific assay, each patient is represented only once to ensure independence. "Independent patients (*n*)" refers to unique individuals. Detailed patient-to-sample mapping is provided in Table S1.

### Isolation of target cells and tissue cells

For cell sorting, peripheral blood samples were collected from 5 JORRP patients (5 mL each) and 13 age-matched healthy children (1–2 mL each) undergoing routine health check-ups, forming three control groups (Table 1). Peripheral blood mononuclear cells (PBMCs) were isolated using Ficoll density gradient centrifugation, and surface staining was performed with antibodies against CD3 and TCR Vβ3 (Table 2). CD3+ TCR Vβ3+ and CD3+ TCR Vβ3− T cells were sorted using a BD FACSAria cell sorter (BD Biosciences) with >95% purity.

**TABLE 2:** Various cell surface and intracellular markers in this study^*a*^

Manufacturer	Antibody	Clone/catalog number
BD Biosciences	Vβ3(PE)	JOVI.3/566432
Beckman Coulter	Vβ20(FITC)	ELL1.4/IM1562
BioLegend	CD3 (BV421)	OKT3/317344
BioLegend	CD3 (APC-Cy7)	HIT3a/300318
BioLegend	CD4 (Percp)	OKT4/317432
BioLegend	CD8 (APC)	RPA-T8/301014
BioLegend	CD8 (APC-Cy7)	SK1/344714
BioLegend	CD45 (BV421)	HI30/304032
BioLegend	CD107a (APC)	H4A3/328620
BioLegend	CD69 (PE-Cy7)	FN50/310912
BioLegend	Granzyme B (PE-Cy7)	QA16A02/372213
BioLegend	IFN-γ (APC)	4S.B3/502512

^*a*^ APC, allophycocyanin; FITC, fluorescein isothiocyanate; PE, phycoerythrin.

Due to the limited space in the pediatric larynx, papilloma collection was challenging. To maximize tissue collection, a custom specimen collection device compatible with a powered microdrill was designed and patented (ZL 2021 2 0760913.9, China). Papillomas were dissociated into single-cell suspensions using mechanical dissociation and enzymatic digestion with a Tumor Dissociation Kit and gentleMACS Dissociators (both from Miltenyi Biotec), following the manufacturer’s protocols.

### HPV virus genotyping detection

In total, 57 JORRP patients underwent HPV testing. Papilloma tissues removed during surgery were used, and DNA from the tissue was extracted using a tissue genomic DNA extraction kit (TIANGEN, DP304) following the instructions. The expression of HPV6 or HPV11 virus in the tissue was detected using fluorescent quantitative PCR (TAKARA, RR820). The primer sequences are as follows: HPV6 Forward: TAGTGGGCCTATGGCTCGTC, HPV6 Reverse: TCCATTAGCCTCCACGGGTG. HPV 11 Forward: GGAATACATGCGCCATGTGG, and HPV11 Reverse: CGAGCAGACGTCCGTCCTCG, where a Ct value >30 indicates a positive HPV virus.

### Flow cytometry

To analyze the 24 Vβ families, PBMCs were stained with the membrane protein antibodies CD3, CD8, and the various anti-Vβ family antibodies included in the IOTest Beta Mark TCR Vβ Repertoire Kit (PN IM3497; Beckman Coulter) according to the manufacturer’s guidelines. Note that CD3^+^CD8⁻ was used as a proxy for CD4^+^ T cells in this initial analysis, without specific gating on CD4. To identify cell surface markers of Vβ3 and Vβ20 T cells, PBMCs were stained with the anti-human CD3, CD4, CD8, Vβ3, Vβ20, CD107a, and CD69 antibodies. The digested single-cell suspension from the papillomas tissue was stained with the anti-human CD45, CD3, CD4, CD8, Vβ3, and Vβ20 antibodies.

To analyze the killing capacity of TCR Vβ3+ T cells and TCR Vβ20+ T cells, PBMCs were first stained with the membrane protein antibodies CD3, CD4, CD8, Vβ3, and Vβ20, then perforated, and finally stained with the anti-human granzyme B (GrzB) and interferon gamma (IFN-γ) antibodies. The lymphocytes were gated using forward- and side-scatter characteristics using a BD Fortessa X20 flow cytometer. At least 20,000 CD3+ lymphocyte events were collected for analysis. The collected data were analyzed by FlowJo v10. Fluorescence-conjugated monoclonal antibodies against the molecules listed in Table 1 were purchased from BioLegend or BD Biosciences.

### Gini-TCR index and calculation of expanded TCR Vβ families

The Gini index is a metric commonly used to characterize income distribution in economic statistics. Given the parallels between the distribution of TCR Vβ families and income distribution, the Gini index can also be applied to the analysis of TCR Vβ repertoires through flow cytometry. This approach has already been utilized in TCR sequencing studies (57, 58) and has recently been introduced as a precise method for analyzing TCR Vβ repertoire data obtained via flow cytometry (40, 59). The TCR (Vβ)-Gini index produces scores that range from low to high, reflecting the distribution of TCR Vβ families from an equal distribution (indicating a broad repertoire or a low score) to an unequal distribution (indicating a skewed repertoire or a high score).

### RNA sequencing and data analysis

Thirteen JORRP papillomas and four paired JORRP blood samples were utilized for RNA microarray analysis. Additionally, eight glossopalatine arch mucosa samples from pediatric patients with tonsil hypertrophy and obstructive sleep apnea (OSA) were included as a disease-unrelated comparative group (Table 1). This tissue was selected as a control surrogate due to its histological similarity (squamous epithelial structure) to laryngeal mucosa, providing a relevant comparison in the absence of ethically accessible healthy pediatric laryngeal tissue.

The RNA microarray analysis of these 25 samples was conducted by OE Biotechnology Co., Ltd. (Shanghai, China) using the Agilent SurePrint G3 Human Gene Expression v3.0 Microarray (8 × 60K, Design ID: 072363). In brief, total RNA was quantified with the NanoDrop ND-2000 (Thermo Scientific), and RNA integrity was evaluated using the Agilent Bioanalyzer 2100 (Agilent Technologies). Sample labeling, microarray hybridization, and washing were performed according to the manufacturer’s protocols. Subsequently, total RNA was reverse-transcribed into double-stranded cDNA, and the synthesized cRNA was labeled with Cyanine-3-CTP. The labeled cRNAs were then hybridized to the microarray, and after washing, the arrays were scanned using the Agilent Scanner G2505C (Agilent Technologies). Raw data were extracted using Feature Extraction software (version 10.7.1.1, Agilent Technologies), and basic analysis was performed using Genespring software (version 14.8, Agilent Technologies). Hierarchical clustering was applied to visualize differentially expressed genes across the samples.

RNA from the sorted CD3+ TCR Vβ3+ T cells was extracted using TRIzol (Invitrogen, Norcross, GA) for RNA sequencing library preparation by the KC Stranded Messenger RNA Library Prep Kit for Illumina (Wuhan Seqhealth, Co, Ltd., Wuhan, China). Pre-amplified complementary DNA molecules were tagged with a unique molecular identifier (UMI) containing eight random bases. Qualified reads were aligned to the reference genome using STAR software (version 2.5.3a; https://github.com/alexdobin/STAR) and quantified with featureCounts (Subread-1.5.1; Bioconductor). DEGs across groups were analyzed using the DESeq2 package in R (Bioconductor). Enrichment analyses, including those from the Kyoto Encyclopedia of Genes and Genomes and Gene Ontology, as well as gene set enrichment analysis for DEGs, were performed in R, employing a corrected *P*-value threshold of 0.05 for statistical significance. TCR usage among the sorted cells was analyzed from the RNA sequencing data using MIXCR software (MiLaboratories) to identify V, D, and J segments, as well as CDR3 sequences.

### TCR sequencing and data analysis

Seven JO-RRP papillomas, 12 JO-RRP blood samples, and 6 healthy control blood samples were used for TCR sequencing. RNA was extracted from more than 2 × 10^6^ frozen lysed PBMCs or 500 mg of frozen tissue using the Magnetic Tissue/Cell/Blood Total RNA Kit (TIANGEN Biotech). RNA concentration and integrity were assessed with Qubit 3.0 (Thermo Fisher Scientific) and the 5300 Fragment Analyzer System (Agilent), respectively. The TCR repertoire was amplified using the KC-Digital TCR Library Prep Kit for Illumina (SeqHealth), based on bias-controlled 5′ RACE technology, utilizing over 500 ng of RNA per sample. UMIs were incorporated during cDNA synthesis to minimize PCR amplification bias and exclude false positives. Three PCR steps were performed for cDNA amplification and barcoding before sequencing on the Illumina Novaseq 6000 system, capturing the CDR3 sequences of both TCR α and β chains.

Using fastp (v0.23.0), clean FASTQ data were generated by removing low-quality sequences. Reads with the same unique identifier (UID) were merged for error correction and duplicate removal. MiXCR software (v3.0.13) was then used to align corrected reads against V, D, and J gene fragments from the IMGT database to extract gene information, rearrangement patterns, and CDR3 sequences. VDJ tools and Immunarch were applied for essential statistics and visualizations. A TCR clonotype was defined by the combination of CDR3 amino acid sequences, V gene fragments, D gene fragments, and J gene fragments. Clonal diversity was assessed using Shannon entropy and the D50 index, where Shannon entropy quantified clone frequency variability, and the D50 index indicated the minimum number of TCR clonotypes representing half of the total TCR reads.

### Statistical analyses

Unless otherwise specified, all flow cytometry, RNA sequencing, and TCR repertoire analyses were performed using one representative sample per patient. In analyses involving paired blood-papilloma comparisons, the samples were explicitly matched within the same individual. The number of independent patients included in each experiment is specified in the corresponding figure legends.

The patient cohort is presented by descriptive statistics for numerical and categorical data. The Student’s *t*-test, one-way analysis of variance (ANOVA) test, Mann-Whitney U test, and Kruskal-Wallis H test were used to compare two or more groups of data. Pearson correlation and the Spearman’s rank coefficient were used for correlation study. A *P*-value of <= 0.05 was regarded as significant. Taking the relatively small sample number in the present study into account, the *P*-value between 0.05 and 0.10 was considered a “trend.” Statistical analyses and data visualization were performed using SPSS (v25.0), GraphPad Prism (v8.0), FlowJo (v10.8.1), RStudio (v4.4.1), and an online platform (https://www.bioinformatics.com.cn).

## Data Availability

A portion of the TCR sequencing data supporting the findings of this study was previously published and has been deposited into the CNGB Sequence Archive (CNSA) of China National GeneBank DataBase (CNGBdb) with accession number CNP0005943. The newly generated RNA and TCR sequencing data sets in the current study have been deposited in the NCBI Gene Expression Omnibus (GEO) under accession number GSE333507.

## SUPPLEMENTAL MATERIAL

Legend Data Set S1 jvi.01080-25-s0001.docx Description of Data Set S1.

Figure S1 jvi.01080-25-s0002.tif Flow cytometric analysis of functional markers on TCR Vβ3+ T cell subsets stratified by HPV genotype.

Data Set S1 jvi.01080-25-s0003.xlsx Detailed information of the 189 identified public TRBV28 clonotypes.

Legend for Figure S1 jvi.01080-25-s0004.docx Description of Fig. S1.

Table S1 jvi.01080-25-s0005.xlsx Detailed clinical data of JORRP patients.

ASM does not own the copyrights to Supplemental Material that may be linked to, or accessed through, an article. The authors have granted ASM a non-exclusive, world-wide license to publish the Supplemental Material files. Please contact the corresponding author directly for reuse.
