# Supplementary material for: Clonal expansion and phenotypic alterations of TCR Vβ3+ T cells in juvenile-onset recurrent respiratory papillomatosis: implications for tumor-associated immunity and chemokine-mediated T-cell trafficking
Source: J Virol. 2026 Jun 2;100(6):e01080-25. doi: 10.1128/jvi.01080-25 (PMC13288611; doi:10.1128/jvi.01080-25)
Supplement: Legend Data Set S1 — Description of Data Set S1. [file jvi.01080-25-s0001.docx]

Supplemental_Data_S1: List of 189 public TRBV28 clonotypes in JORRP patients.

The table provides the CDR3 beta amino acid sequences (column: cdr3aa), V gene segments (column: v), D gene segments (column: d), and J gene segments (column: j) for public clonotypes identified through high-throughput TCR sequencing. Public clonotypes were defined as those present in at least two JORRP patients with a read count of ≥10 and were significantly enriched in papilloma tissues relative to peripheral blood (Fisher's exact test, odds ratio >1, P < 0.05) and in JORRP peripheral blood relative to healthy controls. These clonotypes represent disease-specific expansions potentially associated with HPV-driven immune responses in JORRP.
