# Supplementary figures and images for: Clonal expansion and phenotypic alterations of TCR Vβ3+ T cells in juvenile-onset recurrent respiratory papillomatosis: implications for tumor-associated immunity and chemokine-mediated T-cell trafficking
Source: J Virol. 2026 Jun 2;100(6):e01080-25. doi: 10.1128/jvi.01080-25 (PMC13288611; doi:10.1128/jvi.01080-25)

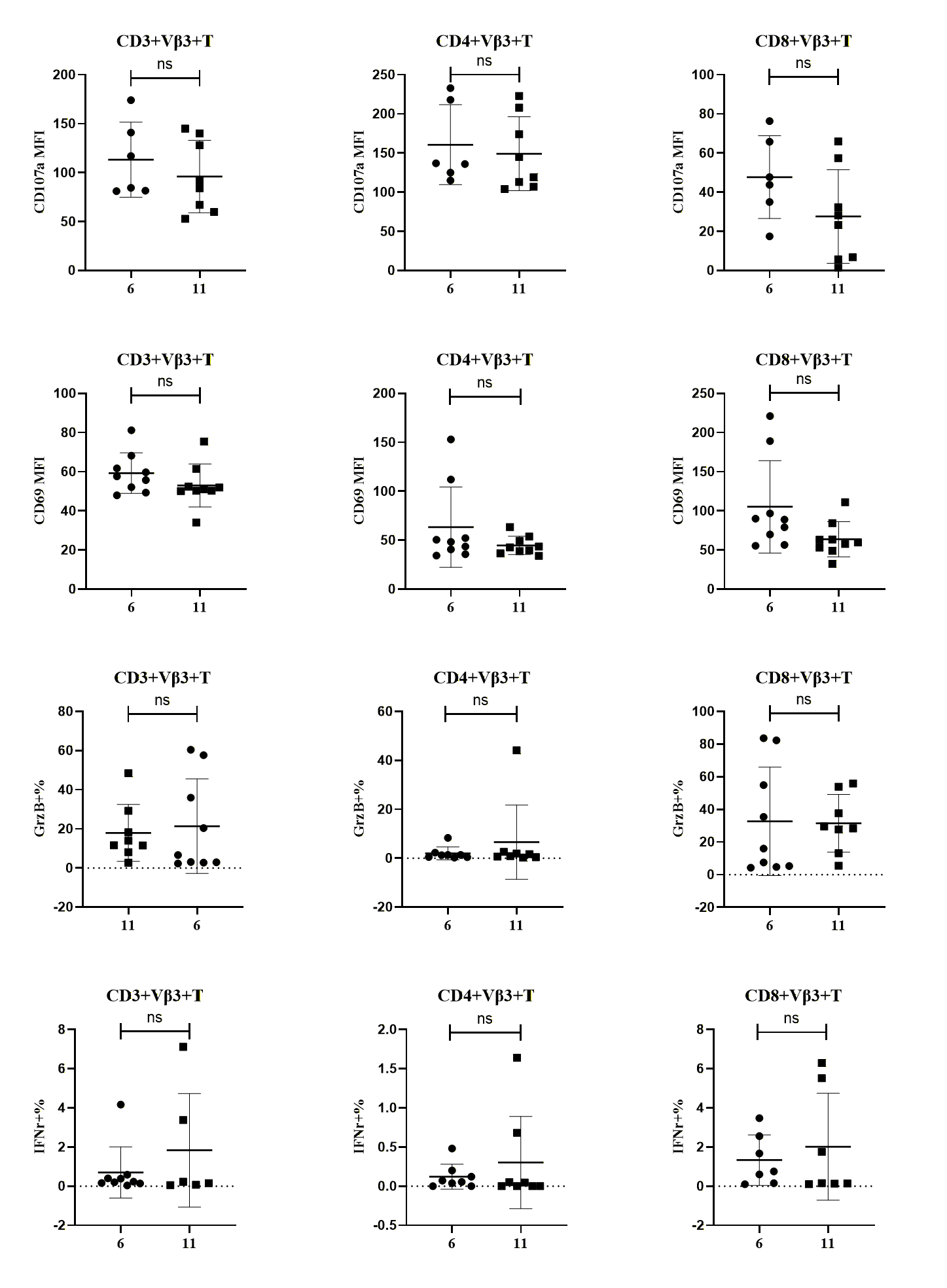

Supplement: Figure S1 — Flow cytometric analysis of functional markers on TCR Vβ3+ T cell subsets stratified by HPV genotype. [file jvi.01080-25-s0002.tif]
