# Supplementary material for: Clonal expansion and phenotypic alterations of TCR Vβ3+ T cells in juvenile-onset recurrent respiratory papillomatosis: implications for tumor-associated immunity and chemokine-mediated T-cell trafficking
Source: J Virol. 2026 Jun 2;100(6):e01080-25. doi: 10.1128/jvi.01080-25 (PMC13288611; doi:10.1128/jvi.01080-25)
Supplement: Legend for Figure S1 — Description of Fig. S1. [file jvi.01080-25-s0004.docx]

**Figure legend:**

Supplemental Figure S1: Flow cytometric analysis of functional markers on TCR Vβ3+ T cell subsets stratified by HPV genotype. Scatter plots depict the expression levels (MFI) of CD107a and CD69, and the percentages of GrzB+ and IFN-γ+ cells within CD3+Vβ3+, CD4+Vβ3+, and CD8+Vβ3+ T cells from JORRP patients infected with HPV6 (n = 10) and HPV11 (n = 11), highlighting the lack of significant differences between the two genotypes. ns: not significant.
